# Supplementary figures and images for: Genetic reprogramming of human amniotic cells with episomal vectors: neural rosettes as sentinels in candidate selection for validation assays
Source: PeerJ. 2014 Nov 18;2:e668. doi: 10.7717/peerj.668 (PMC4243337; doi:10.7717/peerj.668)

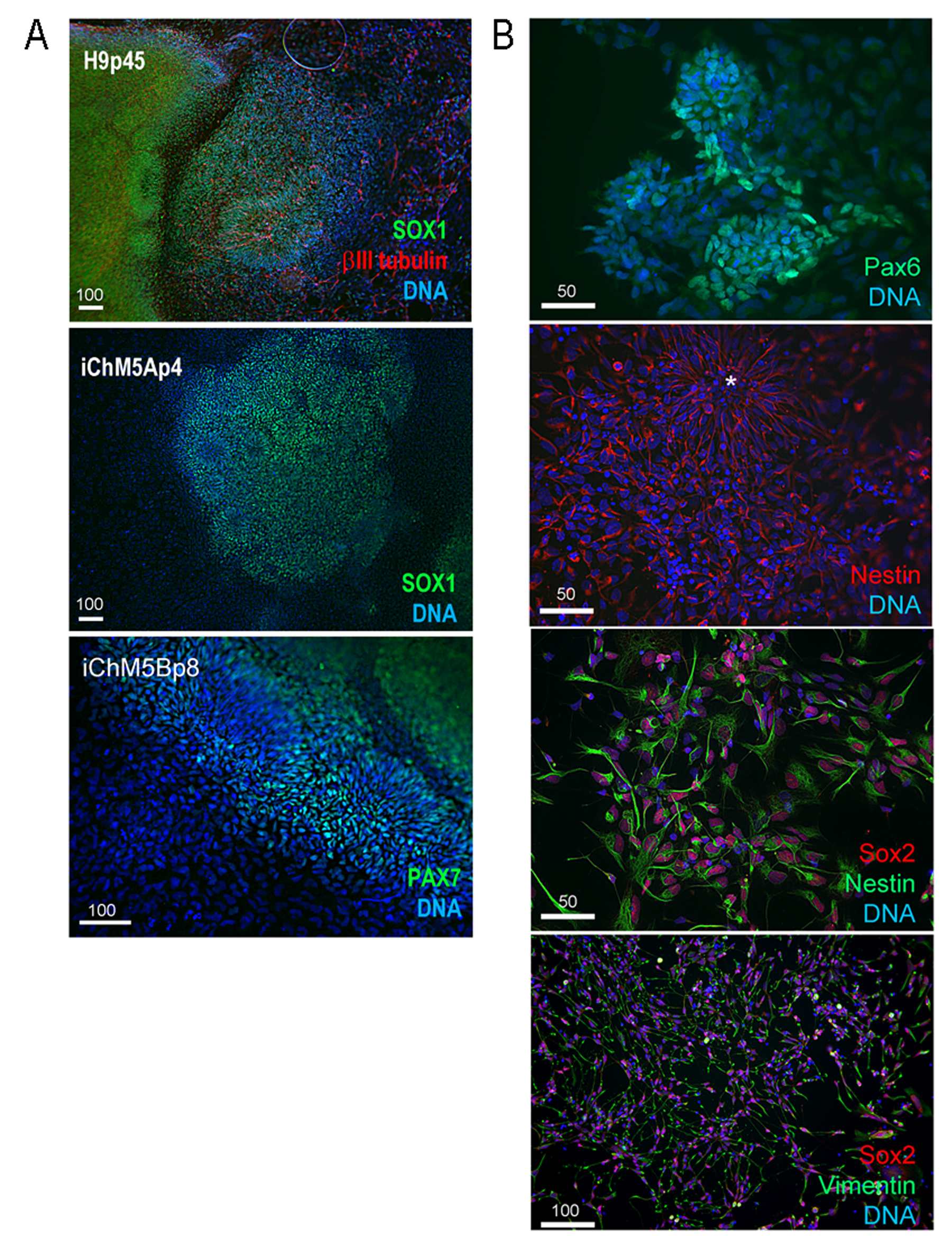

Supplement: Figure S1 — (A). Rosettes. Low magnification image of H9 and iChM5 derived rosettes immunostained as indicated. (B) iChM5Ap4-derived rosettes and NSPs. Dissociated rosettes from candidate colonies were immunostained as indicated. Rosette immunostained for nestin is indicated by asterisk (*). (A,B) Scale bar, in microns as indicated. [file peerj-02-668-s001.png]

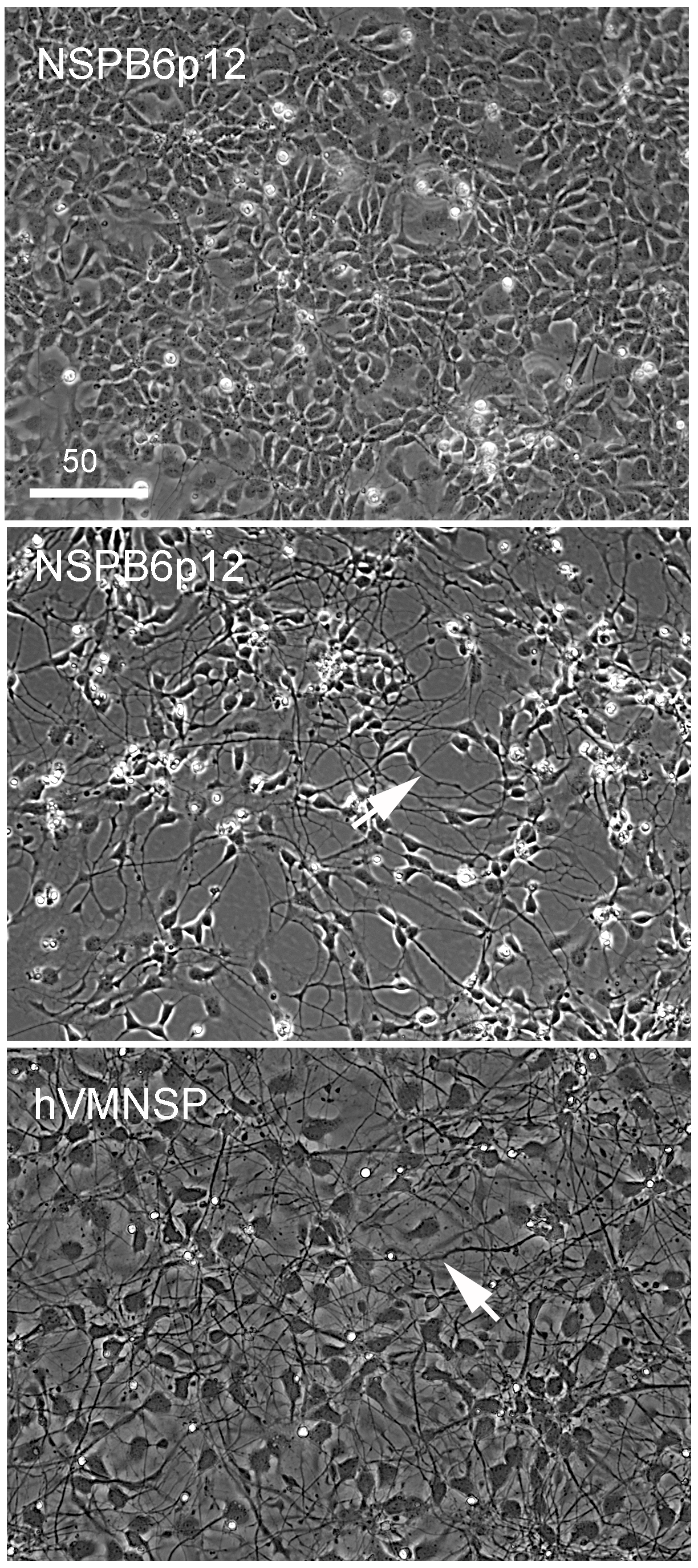

Supplement: Figure S2 — Phase images of NSPB6p12 showing early stage differentiation by withdrawal of mitogens in confluent culture in top image. Middle and bottom images show induced differentiation of NSPB6p12 cells and control hVMNSPs, respectively, at day 7. Representative of presumptive axonal extensions are indicated by arrows. Scale bar, in microns as indicated. [file peerj-02-668-s002.png]

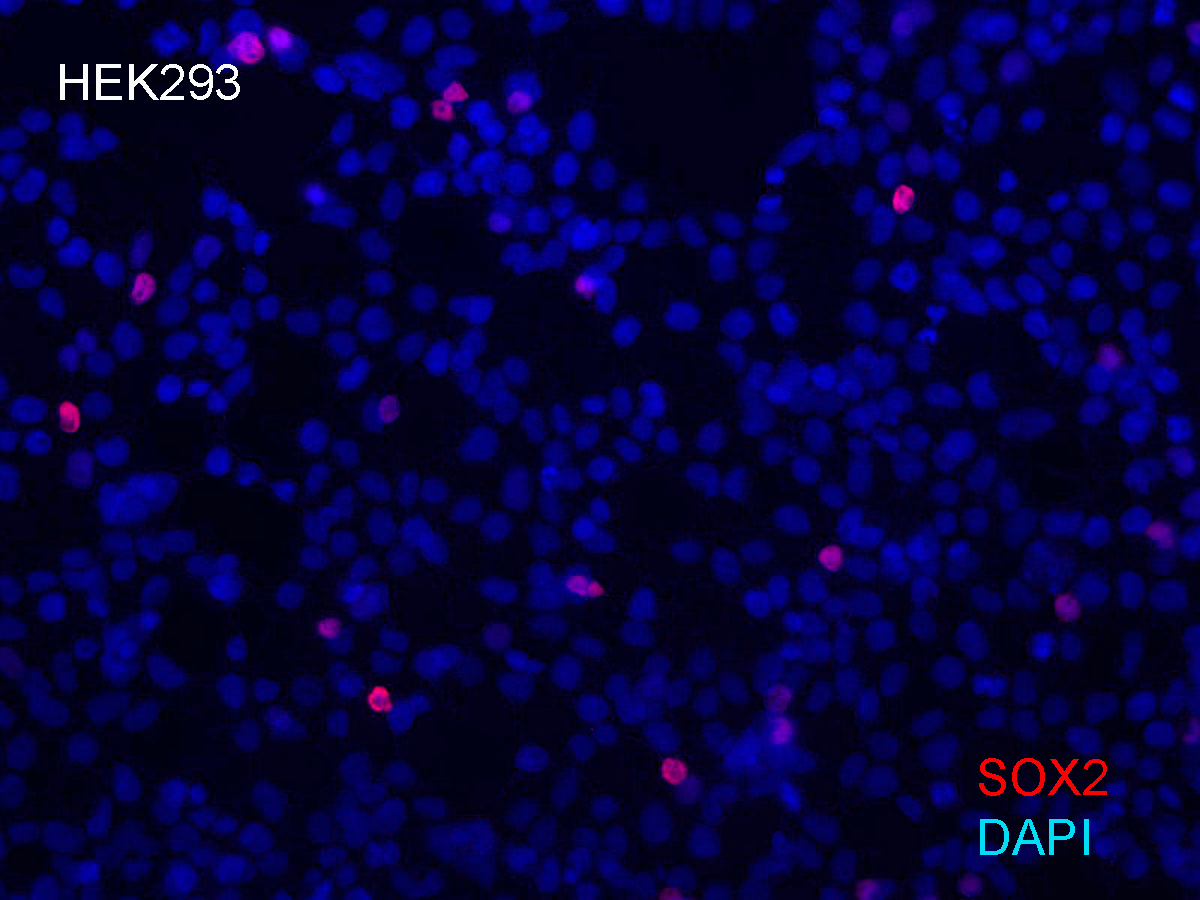

Supplement: Figure S3 — This image show immunodetection of Sox2 in a subset of HEK293 cells that were transfected with the 2 vector combination of episomal plasmids. This image shows the vast majority of cells did not show SOX2 expression, likely because they lacked an episomal vector and did not express an endogenous genes. [file peerj-02-668-s003.png]
